# The steroid-sparing effects of a mycophenolate mofetil-based regimen in the management of immunoglobulin A nephropathy in patients with histologically active lesions: A comparison with a control cohort receiving conventional therapy

**DOI:** 10.1007/s40620-023-01636-6

**Published:** 2023-06-12

**Authors:** Dario Roccatello, Andrea Careddu, Michela Ferro, Carla Naretto, Giacomo Quattrocchio, Roberta Fenoglio, Savino Sciascia

**Affiliations:** University Center of Excellence on Nephrologic, Rheumatologic and Rare Diseases (ERK-Net, ERN-Reconnect and RITA-ERN Member), Nephrology and Dialysis Unit and Center of Immuno-Rheumatology and Rare Diseases (CMID), Coordinating Center of the Interregional Network for Rare Diseases of Piedmont and Aosta Valley (North-West Italy), Department of Clinical and Biological Sciences, University of Turin and San Giovanni Bosco Hub Hospital, ASL Città di Torino, Turin, Italy

**Keywords:** IgA nephropathy, Mycophenolate mofetil, Steroid-sparing

## Abstract

**Introduction:**

While the use of different immunosuppressants has been investigated in immunoglobulin A nephropathy, further investigation is needed to assess the effect of a regimen of mycophenolate mofetil combined with a short course of glucocorticosteroids in the subset of patients with histologically active features. We compared the efficacy and safety of a combined regimen of mycophenolate mofetil and glucocorticosteroids to a conventional regimen of glucocorticosteroids alone in patients with immunoglobulin A nephropathy who have active lesions and major urinary abnormalities.

**Methods:**

This retrospective study involved 30 immunoglobulin A nephropathy patients with active histological lesions, 15 of whom were treated with both mycophenolate mofetil 2 g/day for 6 months and 3 pulses of 15 mg/kg methylprednisolone, followed by a short tapering schedule of oral prednisone. The control group was made up of the remaining 15 clinically- and histologically-matched patients treated with glucocorticosteroids alone according to a validated schedule, i.e., 1 g of methylprednisolone given intravenously for 3 consecutive days, followed by oral prednisone 0.5 mg/kg every other day for 6 months. At diagnosis, all patients had urinary protein excretion > 1 g/24 h and microscopic hematuria.

**Results:**

At the end of the first year of follow-up (30 patients) and after 5 years (17 patients), there were no differences between the two groups in terms of urinary abnormalities and functional parameters. Both regimens achieved a statistically significant decrease in 24-h urinary protein excretion (*p* < 0.001) and a reduction of microscopic hematuria. However, the mycophenolate mofetil-based regimen allowed a cumulative sparing dose of 6 g of glucocorticosteroids.

**Conclusion:**

In this single center study on immunoglobulin A nephropathy patients with active lesions and major urinary abnormalities and at increased risk of glucocorticosteroid-related complications, a mycophenolate mofetil-based regimen demonstrated similar outcomes in terms of complete response and relapse (at 1 and 5 years) compared to a conventional glucocorticosteroid-based protocol, while achieving a consistent reduction of glucocorticosteroid cumulative dose.

**Graphical Abstract:**

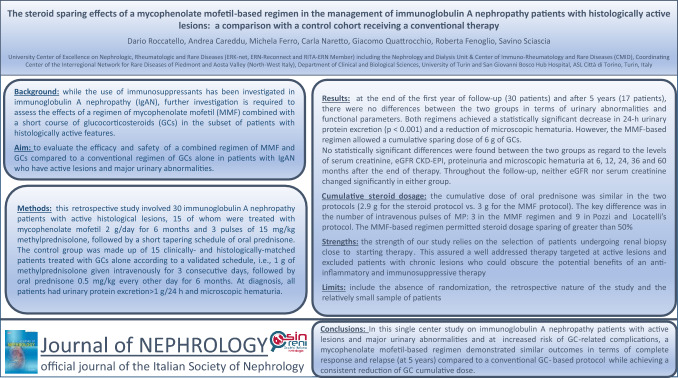

## Introduction

Immunoglobulin A nephropathy (IgAN) is the most prevalent form of primary glomerulonephritis worldwide. Approximately 30% of patients will reach end-stage renal disease within 10–30 years [1]. The best treatment for IgAN has not yet been defined.

Proteinuria is an important predictor of poor outcome. The 2021 Kidney Disease: Improving Global Outcomes (KDIGO) clinical practice guidelines [2] recommend a six-month course of glucocorticosteroid (GC) therapy only in patients with proteinuria > 0.75–1.0 g/day following supportive care with renin-angiotensin system (RAS) blockade for at least 90 days. The use of immunosuppressive drugs is not recommended by KDIGO guidelines, with the exception of Mycophenolate Mofetil (MMF) in Chinese patients [2]. However, the vast majority of open and controlled trials and, disappointingly, even a recent randomized controlled trial-(RCT), analyzing the long-term role of an immunosuppressive regimen in IgAN, included patients with different histological characteristics [3–6].

In previous studies, renal biopsies were not performed at the same interval from the beginning of therapy, despite attempts to match patients according to the mesangial hypercellularity (M), endocapillary hypercellularity (E), segmental glomerulosclerosis (S), and tubular atrophy/interstitial fibrosis (T)—crescents (C) (MEST-C) score. This is a critical point since IgAN is a histologically changing condition over time. To evaluate the effectiveness of any therapeutic approach, a kidney biopsy performed within two months prior to starting therapy should have been required. No histological parameter, including MEST-C score, can accurately determine the impact of any experimental regimen if biopsies are not performed shortly before the start of therapy. The MEST-C score includes active inflammatory (such as mesangial hyperplasia, endocapillary hypercellularity and crescents) and chronic (such as segmental glomerulosclerosis and interstitial fibrosis/tubular atrophy) lesions. While they are considered biomarkers of poor prognosis, chronic lesions are unlikely to improve, regardless of the anti-inflammatory/immunomodulating therapy employed. Inclusion of a substantial proportion of patients with chronic lesions might obscure  the detection of benefit for putatively reversible conditions. Patients with advanced fibrosis would not be expected to improve with any treatment.

Overall, the vast majority of studies [4–14], including a recently published RCT [3], lacked any histological stratification aimed at identifying the cases in which anti-inflammatory/immunosuppressive therapy could actually be effective. Moreover, patients in Rausen’s RCT [3] were treated with an immunosuppressive regimen after a 6-month running course of a RAS-based regimen, which prevented immunosuppressive therapy  from having a timely effect on the flogistic lesions. Therefore, despite several contributions from the literature, the treatment of IgAN patients who are at risk of more rapid progression remains to be established.

The present research is a single-center study on 30 IgAN patients undergoing renal biopsy shortly before treatment start, who showed histological features of active lesions. This study aimed to evaluate the clinical efficacy and safety of an MMF-based regimen compared with a conventional GC-based scheme in this carefully selected sample of IgAN patients.

## Methods

This retrospective study included 30 consecutive adult patients attending our center from 2011 to 2021 with active IgAN defined as follows:

-  > 1 g of proteinuria/24 h; and,
- at least two of the following features in a renal biopsy with > 9 glomeruli performed within two months before therapy start: endothelial hyperplasia, fibrinoid necrosis, crescents and/or abnormal attachment of the glomerular tuft to Bowman’s capsule as sequelae of extra-capillary lesions, and extension of immune reactants to the glomerular capillaries.

### Case identification

**Fig. 1:**
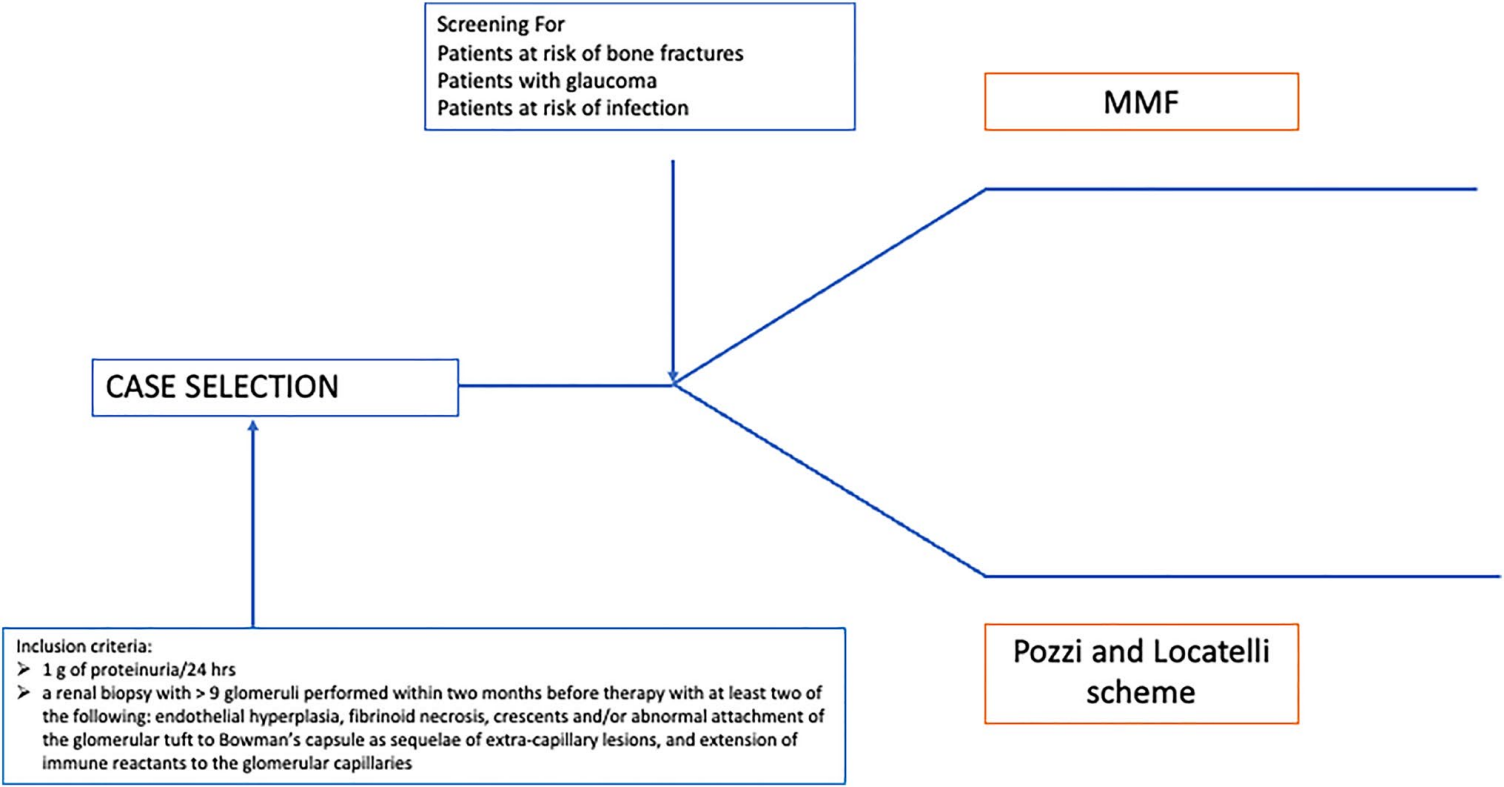
Patient selection flow-chart. Abbreviation: *MMF* mycophenolate mofetil

Patients who were at high risk of GC-related side effects because of the presence of at least one of the following characteristics were treated with MMF (Fig. 1):

- patients at risk of bone fractures or known osteoporosis (according to dual-energy X-ray absorptiometry performed within 3 months before study enrollment);
- glaucoma;
- history or recurrent infection (recurrent infections defined as two or more severe infections in one year, three or more respiratory infections (e.g., sinusitis, otitis, bronchitis) in one year, or the need for antibiotics for two months/year).

### Exclusion criteria

Biopsies were independently scored by two pathologists. Patients with a biopsy showing more than 50% glomerular obsolescence, those with IgA vasculitis or other putative secondary IgAN, active hepatitis, cancer, or a history of immunosuppressive therapies before renal biopsy were excluded.

### Treatment protocol

Patients who met the inclusion criteria and were considered at high risk of GC-related adverse events were treated with a combined schedule of MMF 2 g for 6 months and 3 pulses of methylprednisolone (MP) (15 mg/kg each, maximum 1 g) which was followed by oral prednisone 0.8 mg/kg body weight for 2 weeks, 0.6 mg/kg for another 2 weeks, 0.4 mg/kg for an additional 4 weeks, then tapered by 5 mg every two weeks until discontinuation. Controls were treated with steroids alone according to the Pozzi and Locatelli schedule, which included 1 g of MP for 3 consecutive days at the beginning of the course and again 2 and 4 months later, and oral prednisone at a dose of 0.5 mg/kg every other day for 6 months [4].

All patients received angiotensin converting enzyme inhibitors (ACE-Is) or angiotensin receptor blockers (ARBs) at the maximum tolerated dose during the study.

Serum creatinine, proteinuria and microscopic hematuria (presence of urinary hemoglobin > 0.1 mg/dl), and glomerular filtration rate (as eGFR Chronic Kidney Disease Epidemiology Collaboration [CKD-EPI]) were among the laboratory data that were collected at the time of renal biopsy. This was performed at the 6th and 12th month of follow-up, and, whenever possible, after 2, 3 and 5 years (17 patients).

### Outcomes

Complete response was defined as proteinuria dropping to 0.5 g/day with stable (unchanged or < 25% reduction eGFR) or improved eGFR (primary end point).

Relapse was defined as the reappearance of significant proteinuria, defined as > 1.0 g/day.

Hematuria was defined as detection of ≥ 5 red blood cells/high-power field in centrifuged specimens. Urinary sediment was examined with an automatic system and manually confirmed for the purpose of direct quantification and comparison.

### Statistical analysis

Differences among laboratory data at baseline and during follow-up were analyzed by the t-test for parameters with normal distribution and the Mann–Whitney *U* test for parameters with non-normal distribution. One-way ANOVA was used to compare differences in laboratory data between groups at time 0 and at 6, 12, 24, 36 and 60 months of follow-up.

Fisher’s exact test was also used to calculate corrections and statistical significance. Kaplan–Meier survival analysis was used to investigate differences in therapeutic response between cases and controls. Statistical analyses were carried out using SPSS software (IBM Corporation, NY, USA). Differences were considered statistically significant when 2-sided *p* values were < 0.05.

This study was conducted according to the Piedmont and Aosta Valley ( North-West Italy) legislation for Rare Diseases (N. 1577/UC/SAN of 11.10.2005 based on Regional Government Act 23 April 2007 dealing with Rare Diseases, Systemic Sclerosis RM0091; article. 1: 796 paragraph Z Law number 296 of 2006. Number 5-5740) and the Helsinki Declaration. Each patient provided written consent to participate.

## Results

Thirty patients with primary IgAN, histologically active lesions and proteinuria > 1 g/day were evaluated at 6 months, 1 year and whenever possible at 2, 3 and 5 years after the end of therapy (median 8.92 years, range 1–18). Fifteen of them had been treated with the MMF-based regimen and 15 matched patients with the 6-month Pozzi and Locatelli scheme.

Mean age at the time of therapy was 41 years (range 18–76 years). In the MMF group there were 13 male and 2 female patients (mean age 42 years, SD ± 17), while in the control group there were 9 male and 6 female patients (mean age 39 years, SD ± 14).

Proteinuria was 2.69 ± 1.8 g/day in the MMF cohort and 2.04 ± 1.5 g/day in the control group under steroid-based therapy. Twenty-three (73%) had serum creatinine > 1 mg/dl. Twenty-three (73%) presented with microscopic hematuria at baseline. Mean eGFR CKD-EPI was 65 ± 27 ml/min in the MMF group and 83 ± 31 ml/min in the control group at the time of biopsy (*p* = 0.1).

The mean number of glomeruli at biopsy was 21 (range 9–70) and glomerular sclerosis was present on average in 18% (range 0–50%). Moreover, 25 out of 30 patients had diffuse mesangial proliferation, 27 patients had crescents or synechiae, 8 patients fibrinoid necrosis and 20 patients showed immune reactant (IgA and C3) deposits in the glomerular capillary walls.

The main clinical and histological features of the study and control groups were comparable at baseline (Table 1).

**Table 1:** Demographic, clinical and histological features at baseline

	All patients (*n* = 30)	MMF + steroid (*n* = 15)	Steroid (*n* = 15)	*p* value
Sex: M–F	22–8	13–2	9–6	0.2
Caucasian – Asian	29–1	14–1	15–0	1
Age at biopsy (years)	41 ± 16	42 ± 17	39 ± 14	0.65
Diabetes (N.)	1/30	0/15	1/15	1
Hypertension (N.)	19/30	11/15	8/15	0.4
ACEi/ARB (N.)	15/30	9/15	6/15	0.4
Nephrotic syndrome (N.)	6/30	5/15	1/15	0.1
sCr at diagnosis (mg/dl)	1.42 ± 1.05	1.70 ± 1.30	1.14 ± 0.40	0.14
24 h UPE at diagnosis (g/24 h)	2.36 ± 1.69	2.69 ± 1.8	2.04 ± 1.50	0.31
MH at diagnosis (N.)	23/30	11/15	12/15	0.6
eGFR at biopsy (ml/min/1.73m2)	74 ± 30	65 ± 27	83 ± 31	0.37
# glomeruli (N.)	21 ± 13.5	18 ± 8.9	24 ± 16.2	0.24
% sclerosis	18 ± 14.6	18 ± 13.5	18 ± 15.9	1
M	25/30	12/15	13/15	1
C	27/30	13/15	14/15	1
E	9/30	2/15	7/15	0.1
Fibrinoid necrosis (N.)	8/30	3/15	5/15	0.6
Capillary deposits (N.)	20/30	10/15	10/15	1

*ACEi * ACE-inhibitor, *ARB* angiotensin receptor blocker, *sCr* serum creatinine, *UPE* urinary protein excretion, *MH* microscopic hematuria, *eGFR* estimated glomerular filtration rate (CKD-EPI), *M* diffuse mesangial proliferation, *C* crescents or synechiae, *E* endocapillary proliferation, *N.* number of subjects

**Fig. 2:**
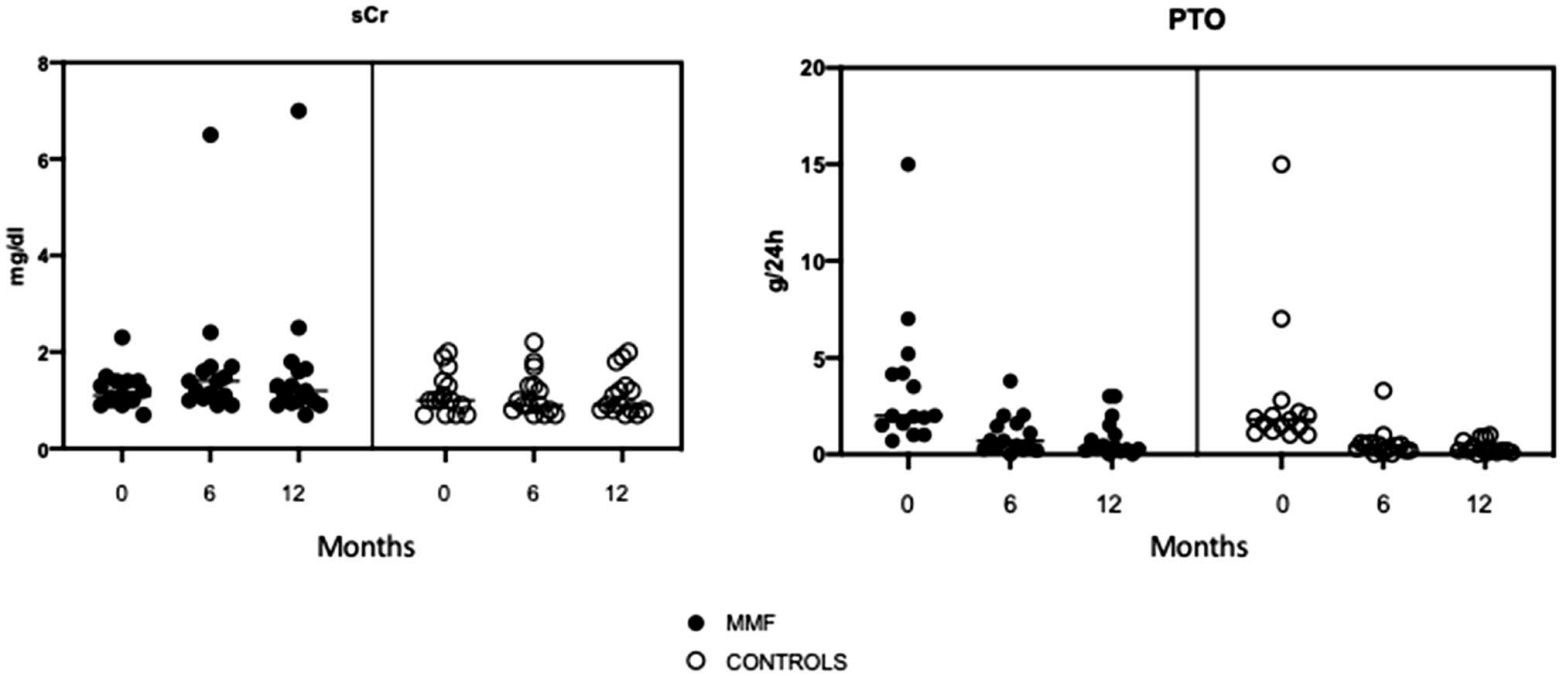
Scatter dot plots showing serum creatinine and proteinuria levels at baseline and during follow-up in both cases and controls

Both the MMF-based regimen and the 6-month steroid scheme (*p* < 0.001) showed a statistically significant decrease in 24-h urinary protein excretion at 12 months and during the subsequent years of follow-up (Fig. 2). In particular, both therapeutic protocols resulted in a statistically significant reduction in proteinuria (*p* < 0.001) at 6 months as compared to baseline data. The primary end point (proteinuria < 0.5 g/day) was achieved in both groups, with a decrease that was quantitatively identical (*p* = 0.56). Incidentally, microscopic hematuria showed a statistically significant reduction at 1 year after the end of therapy in both the MMF-based regimen (*p* = 0.01) and in the steroid group (*p* = 0.02). No statistically significant differences were found between the two groups in regard to levels of serum creatinine, eGFR CKD-EPI, proteinuria and microscopic hematuria at the time of biopsy, as well as at 6 and 12 months after the end of therapy, and at 24, 36 and 60 months. Throughout the follow-up, neither eGFR nor serum creatinine changed significantly in either group.

Adverse events are summarized in Table 2.

**Table 2:** Side effects

	MMF + steroid (*n* = 15, %)	Steroid (*n* = 15, %)
Gastroenterological intolerance (including nausea, diarrhea or gastric discomfort)	2^a^ (13%)	1 (7%)
Steroid-induced diabetes (in patients with no known history of diabetes)	2 (13%)	4 (27%)
Infections	1 community-acquired pneumonia^b^ (7%)	3 UTI^b^ (20%)

*UTI* urinary tract infections

^a^Occurring within 10 days after the beginning of therapy. Patients were switched to mycophenolic acid

^b^No hospitalization required

### Follow-up and relapse rate

Mean follow-up was 18.7 months (range 14–45) for cases and 21.5 months (range 16–56) for controls. No patient started renal replacement therapy during the observation time.

Three patients in the MMF group and one patient in the control group relapsed. Mean time before relapse for patients in the MMF group was 12, 18, and 36 months, respectively, while the patient in the steroid group relapsed after 36 months. The difference between the number of relapses was not statistically significant between the two groups (*p* = 0.282). Patients were treated with the same therapeutic protocol as before and showed complete response.

### Cumulative steroid dose

The cumulative dose of oral prednisone was similar in the two protocols (2.9 g for the steroid protocol vs. 3 g for the MMF protocol), while patients on the combined MMF regimen discontinued GCs two months earlier. The key difference was in the number of intravenous pulses of MP: three in the MMF regimen and nine in the Pozzi and Locatelli protocol. Lastly, the MMF-based regimen permitted steroid dose sparing of more than 50%.

## Discussion

Our study includes patients with major urinary abnormalities and histologic features of active inflammatory lesions treated with either a combined scheme of MMF and GCs, or a GC-based regimen. Study and control regimens showed an equal decrease in proteinuria without changes in serum creatinine and eGFR. Notably, a statistically significant reduction in microscopic hematuria was also found 1 year after the end of therapy in both the MMF group and in the steroid group. The results achieved in our control group were consistent with previous observations [4, 5]. Conflicting outcomes have been observed in relation to MMF over time. Notably, both doses and duration of therapy with MMF differed widely among trials [6–11, 15].

MMF was tested in various glomerulopathies starting in the mid-1990s. Following a few case reports, [12, 13] Chen et al. [6] published the first clinical trial comparing the efficacy of MMF alone vs. oral prednisone. They enrolled 62 Chinese IgAN patients who had urinary protein excretion > 2 and Lee’s grade IV and V at renal biopsy. Thirty-one subjects were treated with MMF for at least 12 months, while the control group received 0.8 mg/kg/day of oral prednisone. Urinary protein excretion showed a more significant reduction in the MMF group at 12 and 18 months of follow up [6].

Furthermore, in a Belgian trial, 21 out of 34 patients were randomized to receive MMF in association with an ACE-I, while 13 patients were given conservative therapy and placebo. No significant differences were observed between the two groups in regard to proteinuria, serum creatinine, inulin clearance and blood pressure when therapy was discontinued 36 months later [7]. Frisch et al*.* enrolled 32 subjects, including 17 patients who received 1 g/day of MMF for one year and 15 who were given placebo. All subjects were treated with ACE-Is or ARBs. Only 6 patients in the MMF group and 5 in the control group completed 12 months of therapy. Analyses were carried out as intention-to-treat, and patients were selected among those who had a high degree of glomerular sclerosis [8], a condition that is unlikely to respond to immunosuppressive agents.

Two other RCTs are worth considering: a Chinese study [9, 10] and Hogg’s study [11].

The Chinese study enrolled 40 patients [9], twenty of whom received 2 g/day of MMF for 6 months, while the others were given placebo. Inclusion criteria were urinary protein excretion > 1 g/day despite treatment with ACE-Is/ARBs, serum creatinine < 3.5 mg/dl, and grade II–IV histological damage, according to the Haas classification. Mean time between biopsy and therapy was very long. The primary endpoint was remission of proteinuria at 18 months. Sixteen patients in the MMF group and six in the placebo group reached the primary endpoint after 1.5 years. Urinary protein excretion was significantly lower among patients who received immunosuppressive therapy, while eGFR remained unchanged [9]. Interestingly, the difference in proteinuria favoring the MMF group was lost after 6 years of follow up, but over the long term, patients who had been treated with MMF had better renal survival [10].

In Hogg’s study [11], 52 patients were enrolled, with 25 receiving 2 g/day of MMF and 27 given placebo. The intention was to administer the therapy for 12 months, but only 13 subjects in the MMF group and 15 in the placebo group completed the trial due to lack of clinical benefits. The inclusion criteria for this study did not take into account the histological lesions [11]. A more recent contribution, which deals with the use of MMF in monotherapy, enrolled 18 patients who were administered MMF on average for 28 months. Each patient was given RAS blockers unless contraindicated. At the time of the diagnostic biopsy, mean serum creatinine was 1.1 mg/dl and mean urine protein creatinine ratio was 146 mg/mmol. The study aimed at searching for histological changes at a protocol biopsy 24 months after the beginning of therapy. A significant improvement in the mean percentage of glomeruli showing endocapillary hypercellularity and cellular/fibro-cellular crescents was detected [15].

In summary, among the most representative studies dealing with the use of MMF for IgAN, three demonstrated some efficacy and three a lack of clinical benefits (Table 3). Notably, a meta-analysis that included the 5 RCTs described above emphasized that patient ethnicity seemed to be a factor in modulating clinical responses [14].

**Table 3:** Studies on the use of MMF in monotherapy

Study	Type of study	Sample size	Patient characteristics	Regimen	Follow-up	Efficacy
Chen et al*.* [6]	RCT	62 (31 cases, 31 controls)	Severe IgAN, Lee grade IV-V, proteinuria > 2 g/24 h	MMF vs prednisone	12 months	Lower levels of proteinuria in the MMF group
Maes et al*.* [7]	RCT	34 (21 cases, 13 controls)	CKD, advanced histological lesions	MMF vs placebo	36 months	No
Frisch et al*.* [8]	RCT	32 (17 cases, 15 controls)	Severe CKD, advanced histological lesions	MMF vs placebo	24 months	No
Tang et al*.* [9, 10]	RCT	40 (20 cases, 20 controls)	Moderate histological lesions	MMF vs placebo	60 months	The primary end point of reduction of proteinuria by 50% or more over entry level was observed significantly more frequently in the MMF arm. Better renal outcomes at 60 months in MMF-treated patients
Hogg et al*.* [11]	RCT	44 (22 cases, 22 controls)	UPCR ≥ 0.6–0.8 g/g, no histological criteria	MMF vs placebo	6 months	No
Beckwith et al*.* [15]	Retrospective	18	Endocapillary hypercellularity	MMF	24 months	Regression of hypercellularity, stable clinical parameters

The effects of the combined treatment of MMF and steroids have been described in a few studies (Table 4). The therapeutic schedule we used in the present study was firstly applied in a cohort of eight patients who had urinary protein excretion > 1 g/day, persistent microscopic hematuria, renal failure (mean serum creatinine 1.6 mg/dl) and a renal biopsy with florid histological changes. All 8 subjects had hypertension and were being treated with ACE-Is. Mean serum creatinine, proteinuria and microscopic hematuria levels were significantly reduced 6 months after the beginning of treatment. Significant differences were observed in proteinuria when comparing baseline data with those reported at the end of the follow-up (on average 51 months later) [16]. Liu et al*.* enrolled 84 subjects, 42 of whom were treated with MMF and prednisone, while the other 42 were treated with cyclophosphamide and prednisone. Both protocols were effective, but patients treated with MMF had better outcomes and fewer side effects. Moreover, compared to baseline values, urinary protein excretion and microscopic hematuria were significantly lower in patients given MMF [17]. More recently, Hou et al*.* enrolled 176 patients, 87 of whom were treated with MMF and steroids and 89 with steroids alone but at a higher dose. Inclusion criteria were diagnostic renal biopsy within 1 month prior to enrollment, proteinuria ≥ 1 g/day, and eGFR > 30 ml/min. At least one of the following histological lesions had to be present: cellular and fibro-cellular crescents involving 10–50% of glomeruli, endocapillary hypercellularity and glomerular necrosis, while tubular atrophy/interstitial fibrosis had to be < 50%. Six months after the beginning of therapy, 61 patients in the MMF group and 64 in the steroid group had complete remission (i.e., undetectable proteinuria and serum creatinine levels < 25% above baseline). Twelve months after the start of therapy, 60 patients in the MMF group and 61 in the steroid group had complete or partial remission. Total adverse event rates did not differ between the MMF and steroid groups, but those who were treated with corticosteroids alone had a higher incidence of Cushing syndrome and diabetes [18].

**Table 4:** Studies on the combined use of MMF and steroids

Study	Type of study	Sample size	Patient characteristics	Therapy	Follow up	Efficacy
Roccatello et al*.* [16]	Retrospective	8	Florid inflammatory lesions	MMF + steroids	24–90 months	Significant reduction in proteinuria and hematuria
Liu et al*.* [17]	RCT	84 (42 case group, 42 control group)	Lee grade III-IV, proteinuria > 1 g/day	MMF + steroids vs steroids alone	18 months	Significant reduction in proteinuria in the MMF group
Hou et al*.* [18]	RCT	176 (87 case group, 89 control group)	Florid inflammatory lesions	MMF + steroids vs steroids alone	12 months	Significant reduction in proteinuria

Histological data are needed in order to select patients who might potentially benefit from immunosuppressive treatment. Some lesions (endocapillary hypercellularity, florid or fibroepithelial crescents and fibrinoid necrosis) have proven to be reversible after immunosuppressive therapy.

Several studies have highlighted the prognostic value of endocapillary hypercellularity and its ability to predict not only the progressive loss of renal function but also the response to immunosuppressive therapy. Beckwith et al*.*, Hou et al*.*, Hotta et al*.* and Shen et al*.* reported improvement or disappearance of endocapillary hypercellularity following therapy in repeat biopsy [15, 18–20]. Shoji et al*.* also reported regression of the crescents after therapy [21]. Some of these studies, together with Liang’s report, also point out the reduction of fibrinoid necrosis [22].

The results of the present study are consistent with those of Hou et al*.* [18]. Proteinuria significantly dropped in each treatment group after 12 months of follow up. Neither protocol was superior in achieving remission. Renal function did not decrease as compared to baseline. Microscopic hematuria also dropped significantly, just as it did in Liu’s study [17].

Limitations include the absence of randomization, the retrospective nature of the study, the heterogeneity in the length of follow-up (5 years in 17 patients), and the relatively small sample size. Similarly, we acknowledge that previous experience (e.g., the STOP IgAN trial [23]) supports the observation that conservative therapy alone can result in a reduction in proteinuria to less than 0.75 g/day in nearly 30% of cases, and that the ACEi/ARB regimen may have introduced an additional benefit. However, the severity of the histological features of our patients makes this possibility unlikely.

The strength of our study lies in the selection of patients undergoing renal biopsy shortly before starting therapy. This assured that therapy was targeted at active lesions and excluded patients with chronic lesions who could obscure the benefits of an anti-inflammatory and immunosuppressive therapy.

## Conclusions

The present single-center study involving IgAN patients with histologically active lesions showed that an MMF-based regimen was as effective as the validated scheme of steroids recommended by the KDIGO clinical practice guidelines. However, it showed substantial steroid-sparing effects, and suggests an alternative strategy for reducing steroid-related morbidity in patients with metabolic syndrome, diabetes risk or severe osteoporosis. Notably, benefits were confirmed at 5 years of follow up.

In an attempt to conciliate controversial results from differently designed studies, the 2021 KDIGO Guidelines concluded that MMF may be used as a glucocorticoid-sparing agent only in Chinese patients [2]. While observational studies in larger samples of patients and randomized controlled trials are needed to validate our results, MMF could also have a role in Caucasian patients.

## Data Availability

Data are available from the Corresponding Authors upon reasonable request.
